# Supplementary material for: Seminoma and Embryonal Carcinoma Footprints Identified by Analysis of Integrated Genome-Wide Epigenetic and Expression Profiles of Germ Cell Cancer Cell Lines
Source: PLoS One. 2014 Jun 2;9(6):e98330. doi: 10.1371/journal.pone.0098330 (PMC4041891; doi:10.1371/journal.pone.0098330)
Supplement: File S1 — ZIP file containing DMRforPairs output for significant regions. Please start from the html files. (ZIP) [file pone.0098330.s008.zip › figures/40988.pdf]

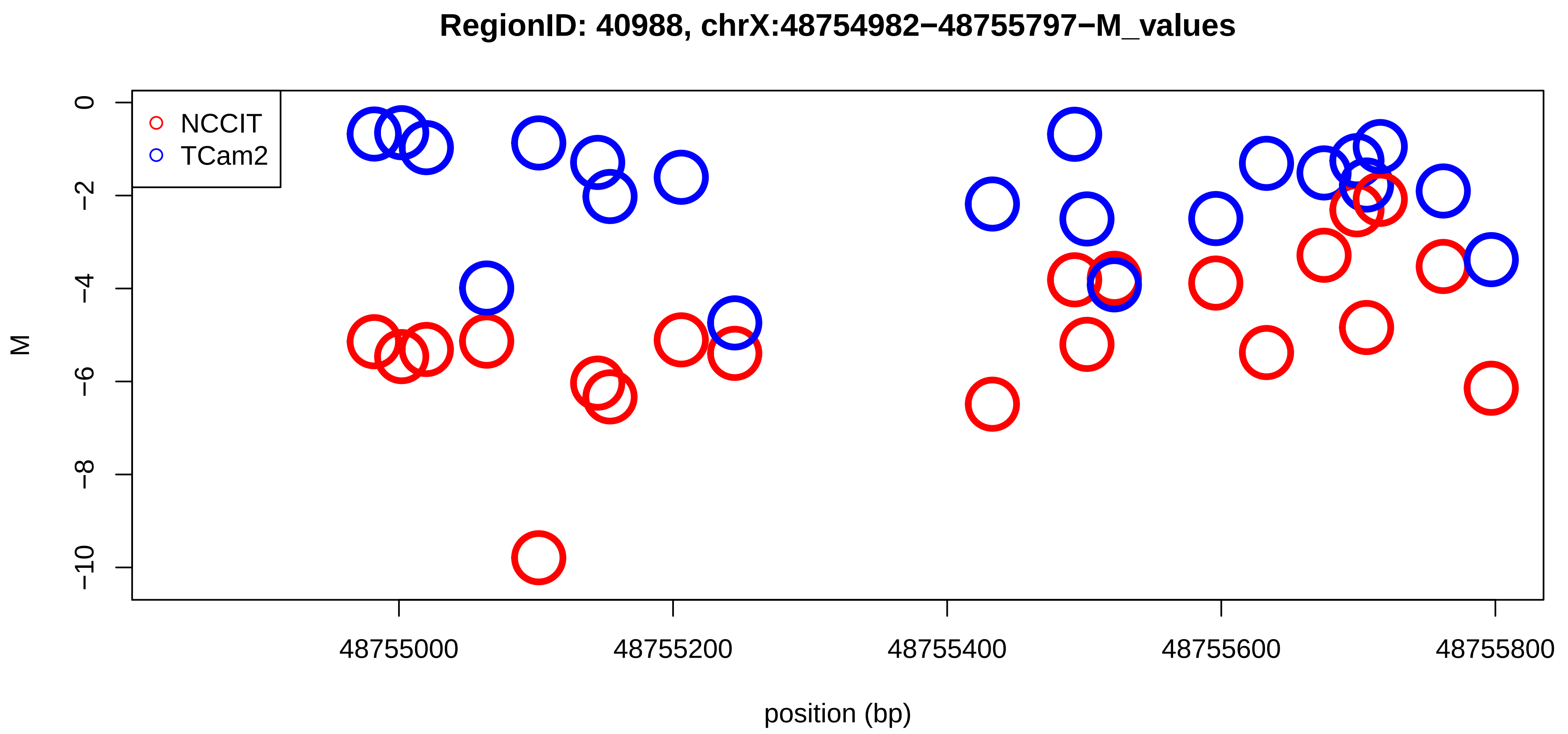
RegionID: 40988, chrX:48754982−48755797−M_values
NCCIT
TCam2
M
0
−2
−4
−6
−8
−10
48755000
48755200
48755400
48755600
48755800
position (bp)

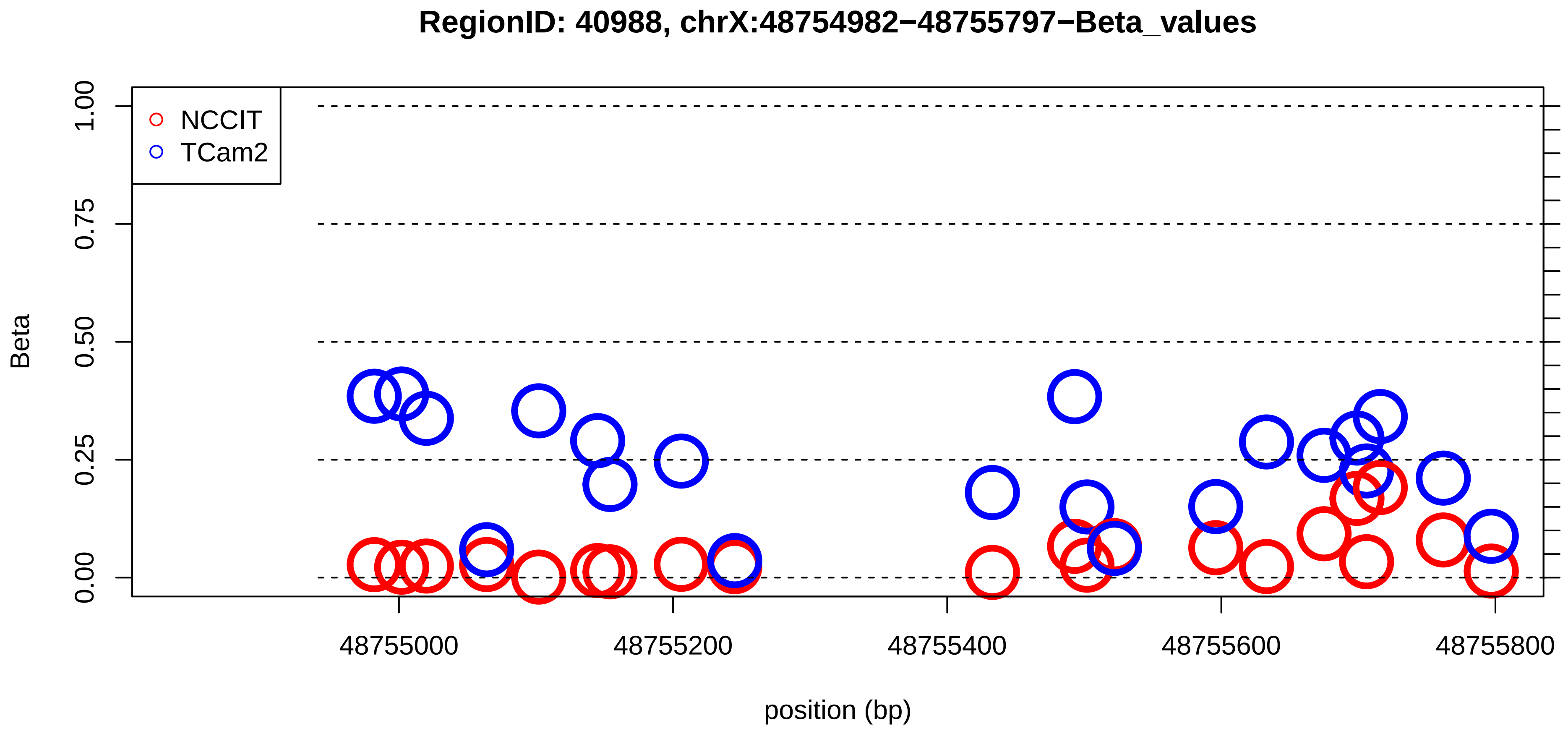
RegionID: 40988, chrX:48754982−48755797−Beta_values
NCCIT
TCam2
Beta
0.00
0.25
0.50
0.75
1.00
48755000
48755200
48755400
48755600
48755800
position (bp)

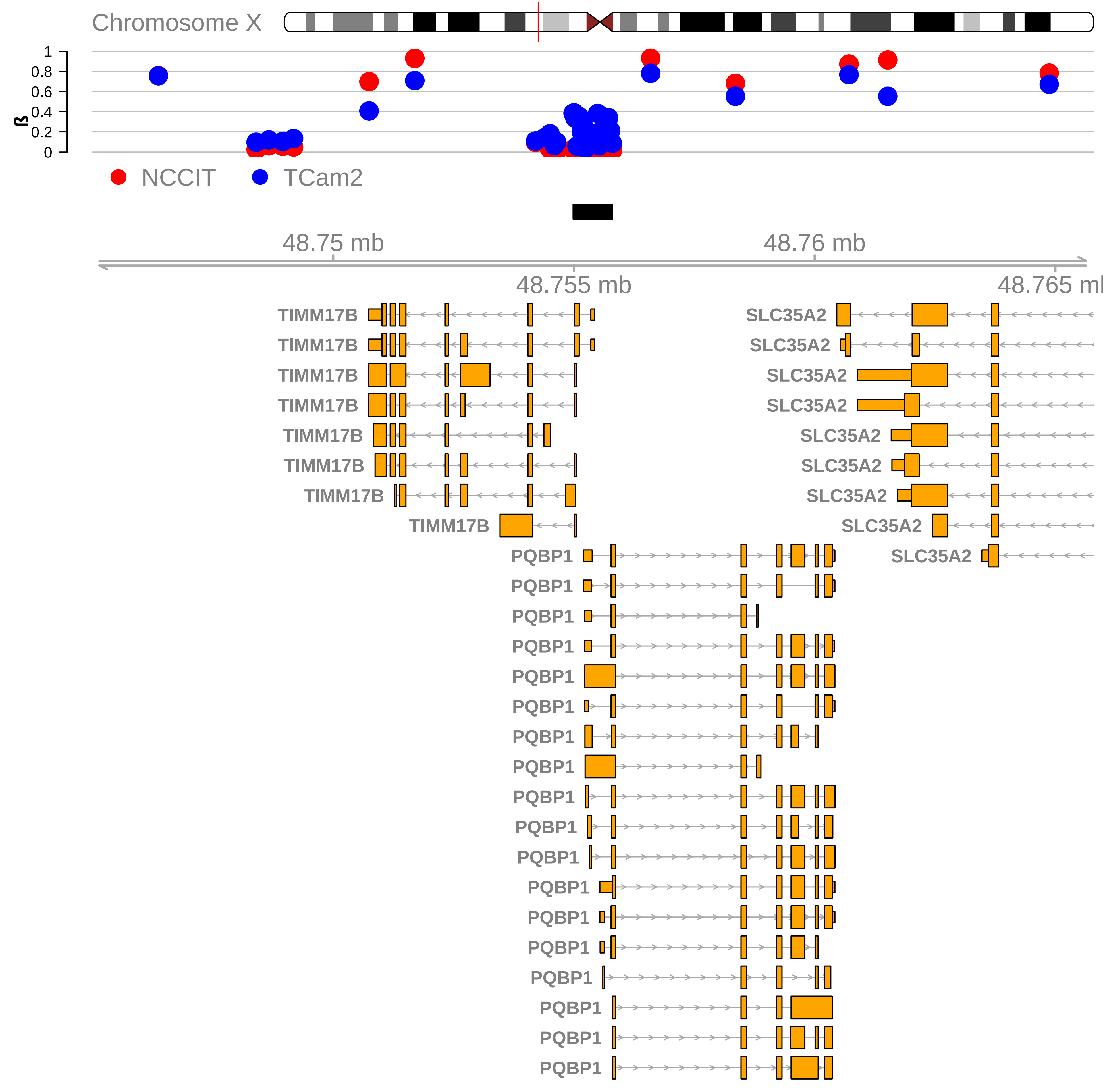

Chromosome X
ß
1
0.8
0.6
0.4
0.2
0
NCCIT
TCam2
48.75 mb
48.76 mb
48.755 mb
TIMM17B
SLC35A2
PQBP1
